# Brucella orchitis: A case report on a rare cause of scrotal abscess leading to orchiectomy

**DOI:** 10.1016/j.eucr.2025.103116

**Published:** 2025-07-04

**Authors:** Behnam Dejman, Fereshteh Rahdan, Majid Dezhman, Dariush Rahdan, Ahmad Movahedpour, Fatemeh Salahpour-Anarjan

**Affiliations:** a Department of Urology, Shahid Beheshti University of Medical Sciences, Tehran, Iran; b Department of Medical Genetics, Faculty of Medicine, Ahvaz Jundishapour University of Medical Sciences, Ahvaz, Iran; c Department of Medical Biotechnology, Faculty of Advanced Medical Sciences, Tabriz University of Medical Sciences, Tabriz, Iran; d Faculty of Medicine, Jahrom. University of Medical Sciences, Jahrom, Iran; e Faculty of Medicine, Fasa University of Medical Sciences, Fasa, Iran; f Department of Medical Biotechnology, School of Advanced Medical Sciences and Technologies, Shiraz University of Medical Sciences, Shiraz, Iran; g Department of Medical Nanotechnology, Faculty of Advanced Medical Sciences, Tabriz University of Medical Sciences, Tabriz, Iran

**Keywords:** Case report, Brucellosis, Scrotal abscess, Orchiectomy

## Abstract

Brucellosis, a zoonotic infection common in Iran, rarely causes genitourinary complications such as orchitis. We report a 65-year-old Persian cattle farmer with brucellosis who developed a testicular abscess despite antibiotic therapy. Initial treatment with rifampin and doxycycline resolved systemic symptoms, but scrotal pain prompted ultrasound, revealing a right testicular abscess. Orchiectomy was performed due to extensive necrosis, with histopathology confirming necrotizing brucellar orchitis. Post-surgical levofloxacin and clindamycin prevented recurrence at six months. This case highlights the need to consider Brucella orchitis in endemic regions when scrotal pathology persists, as advanced cases may require surgical intervention.

## 1 Introduction

Brucellosis, a zoonotic infection caused by Gram-negative coccobacilli, is a notable public health issue in endemic regions like Iran, often transmitted via unpasteurized dairy or animal contact.^1^^,^^2^ Though typically presenting with systemic symptoms such as fever and musculoskeletal pain, genitourinary involvement, particularly orchitis or epididymitis, is uncommon. Rarer still are scrotal complications like abscess formation, which can mimic testicular malignancy or torsion, complicating diagnosis and management.^3^

We report an unusual case of Brucella orchitis in a 65-year-old Persian male cattle farmer, progressing to a scrotal abscess despite antibiotic therapy, ultimately requiring orchiectomy. This case highlights the need to include brucellosis in the differential diagnosis of scrotal pathology in endemic areas, especially when systemic symptoms precede genitourinary findings. Through this report, we aim to detail the clinical progression, diagnostic approach, and management challenges of this rare brucellosis presentation.

## 2 Case presentation

A 65-year-old Persian male cattle farmer presented with low back pain, chills, and fever. He was evaluated by an infectious disease specialist, where laboratory findings showed leukocytosis (white blood cell count [WBC] 21,000/mm^3^), thrombocytosis (platelet count 550,000/mm^3^), elevated C-reactive protein (CRP +4), and an erythrocyte sedimentation rate (ESR) of 65 mm/hour. Serologic tests confirmed brucellosis (Wright titer 1:2560, Widal titer 1:160). Blood and urine cultures were negative. He received oral rifampin (300 mg every 8 hours) and doxycycline (100 mg every 12 hours) for four weeks, resulting in improved laboratory values (WBC 12,000/mm^3^, platelets 250,000/mm^3^, CRP +1, ESR 40 mm/hour, Wright 1:80) and resolution of systemic symptoms. However, he subsequently developed right-sided scrotal pain and was referred to urology.

**Fig. 1:**
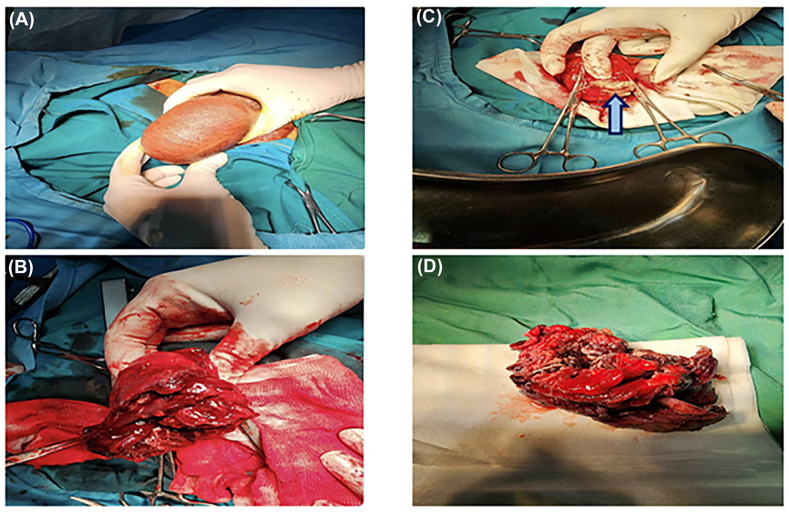
(**A**) Right hemiscrotal swelling. (**B**) Intraoperative view of the necrotic right testis and inflamed spermatic cord. (**C**) Remnant seminiferous tubules attached to the tunica albuginea (arrow). (**D**) Orchiectomy specimen showing the necrotic testis within the tunica vaginalis.

**Fig. 2:**
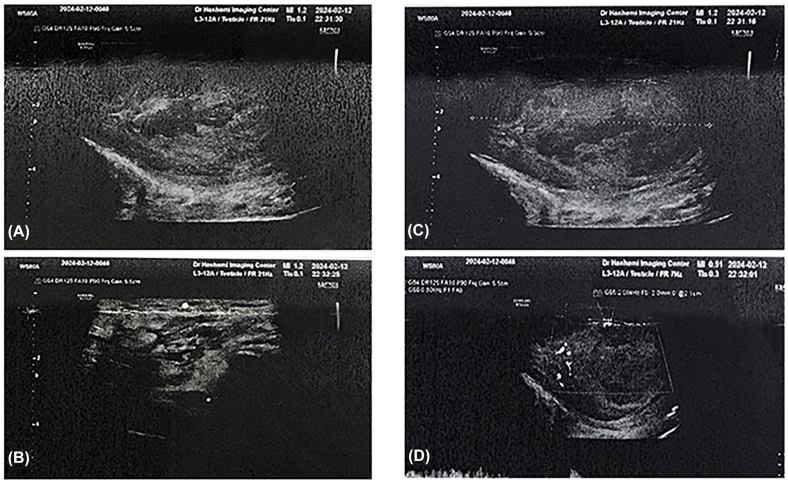
(**A, C**) Ultrasound of the enlarged right testis with hypoechoic areas suggesting abscess formation. (**B**) Thickened spermatic cord with soft tissue edema. (**D**) Peripheral vascularity indicating necrosis.

On examination, the right hemiscrotum was swollen and tender. Scrotal ultrasound revealed an enlarged right testicle (66 × 39 mm) with heterogeneous echotexture and hypoechoic areas (52 × 60 mm), suggestive of an abscess (Fig. 1, Fig. 2). The spermatic cord appeared thickened with vascular congestion. Due to extensive testicular necrosis, a right orchiectomy was performed under spinal anesthesia via a median raphe incision. Intraoperative findings included purulent drainage and a necrotic testis, which was excised, followed by saline irrigation and placement of a Penrose drain. Intraoperative samples were sent for microbial culture; however, no organisms were isolated, likely due to prior antibiotic therapy.

**Fig. 3:**
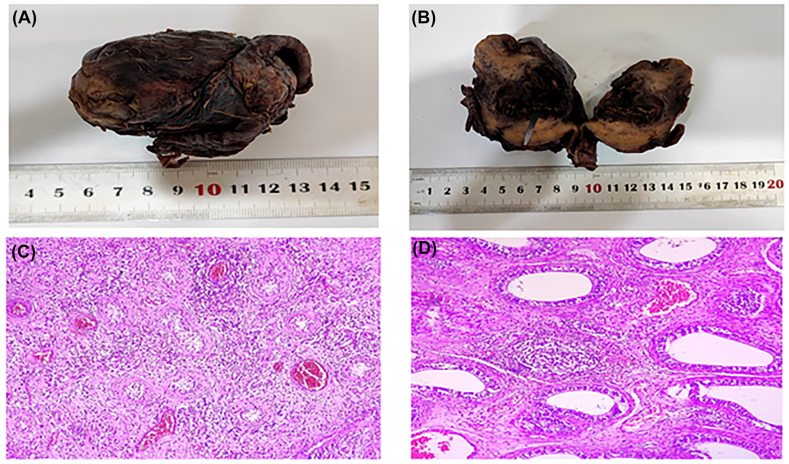
(**A**) Nodular enlargement of the testis. (**B**) Orchiectomy specimen with fibrino-purulent exudate on the cut surface. (**C**) Histopathology showing lymphocyte and plasma cell infiltration around atrophic seminiferous tubules. (**D**) Non-caseating granulomas and chronic inflammation in the epididymis.

Histopathology confirmed necrotizing brucellar orchitis with lymphocyte and plasma cell infiltration, atrophic seminiferous tubules, and non-caseating granulomas in the epididymis (Fig. 3). Postoperatively, the patient received oral levofloxacin (750 mg daily) and clindamycin (300 mg every 6 hours) for two weeks. Follow-up at six months, including blood tests and ultrasound, showed no recurrence (Table 1).

**Table 1:** Timeline of the patient care.

Stage	Timeline (day)	Details
**Diagnosis**	0	Brucella orchitis confirmed (Wright titer 1:2560, Widal 1:160)
**Antibiotic treatment**	1–28	Rifampin 300 mg every 8 hours; Doxycycline 100 mg every 12 hours
**Orchiectomy**	29	Right orchiectomy for testicular abscess
**Post-surgical antibiotics**	29–42	Levofloxacin 750 mg daily; Clindamycin 300 mg every 6 hours
**Follow-up**	43–180	No recurrence or complications at 6 months

## 3 Differential diagnosis

Differential diagnoses included testicular torsion, malignancy, and infectious epididymo-orchitis. Torsion was unlikely due to preserved vascularity on ultrasound and the absence of spermatic cord twisting. Malignancy was considered given the solid nodular enlargement and heterogeneous echotexture; however, histopathology revealed chronic inflammation, lymphocyte and plasma cell infiltration, non-caseating granulomas, and fibrino-purulent exudate, confirming necrotizing brucellar orchitis (Fig. 3). The presence of granulomas and the patient's occupation in an endemic region further supported the diagnosis.

## 4 Outcome and follow-up

Following right orchiectomy, the patient received oral levofloxacin (750 mg daily) and clindamycin (300 mg every 6 hours) for two weeks. He was hospitalized for one week with regular blood tests and ultrasound monitoring. At six months, the patient remained asymptomatic with no signs of recurrence or complications (e.g., chronic pain or sexual dysfunction), as confirmed by clinical evaluation and imaging (Table 1).

## 5 Discussion

Brucellosis, a zoonotic infection prevalent in endemic regions like Iran, typically presents with systemic symptoms, but genitourinary involvement such as orchitis is rare, affecting less than 10 % of cases.^3^ Scrotal abscesses are even less common, with few reported instances.^4^^,^^5^ This case of Brucella orchitis progressing to a scrotal abscess despite antibiotics highlights its diagnostic and therapeutic complexity.

The condition's presentation often mimics testicular malignancy or torsion, delaying accurate diagnosis. Our patient's nodular enlargement and heterogeneous echotexture resembled findings by Reisman et al.^6^ and Turhan et al.,^7^ where granulomatous orchitis prompted orchiectomy due to suspected tumors.^1^ Unlike those cases, systemic brucellosis history and serologic confirmation clarified the infectious cause here, emphasizing brucellosis as a key differential in endemic areas.

Standard treatment involves rifampin and doxycycline.^8^ In our case, despite apparent compliance and improvement in systemic symptoms, the disease progressed locally. This may be explained by the formation of an abscess and extensive tissue necrosis, which can limit antibiotic penetration and reduce treatment efficacy. Moreover, given the presence of purulent drainage and the risk of superinfection with Gram-positive or Gram-negative bacteria, the postoperative antibiotic regimen was changed to levofloxacin and clindamycin based on infectious disease consultation and local antibiogram patterns. Dede et al.^9^ reported success with antibiotics alone for small abscesses, yet our case required orchiectomy due to extensive necrosis, consistent with Bapir et al.^3^ and Erkoç et al.^10^ where surgery was necessary for advanced complications. This variability stresses the need for early intervention tailored to disease severity.

This report adds to the sparse literature on Brucella orchitis by documenting its progression to abscess and the role of surgery in severe cases. Clinicians in endemic regions should suspect brucellosis in scrotal pathology, especially with relevant exposure, to optimize outcomes and potentially avoid orchiectomy.

## 6 Conclusion

This case highlights the importance of including Brucella orchitis in the differential diagnosis of testicular abscesses in endemic regions like Iran, where systemic brucellosis may precede genitourinary complications. The progression to scrotal abscess despite antibiotic therapy underscores the potential need for orchiectomy in advanced cases. Clinicians should maintain a high index of suspicion for brucellosis in patients with scrotal symptoms and relevant exposure history, prioritizing early serologic testing and imaging to optimize management and potentially avoid surgical intervention.

## CRediT authorship contribution statement

**Behnam Dejman:** Conceptualization. **Fereshteh Rahdan:** Writing – original draft, Visualization, Validation, Supervision. **Majid Dezhman:** Resources. **Dariush Rahdan:** Data curation. **Ahmad Movahedpour:** Formal analysis. **Fatemeh Salahpour-Anarjan:** Writing – review & editing, Project administration.

## Patient consent

Written informed consent was obtained from the patient for the publication of this case report.

## Ethical approval

Our institution does not require ethical approval to report individual cases.

## Interest to declare

The authors declare no conflicts of interest.

## Funding

No funding was received for this work.
